# Piezoelectric Scaffolds as Smart Materials for Bone Tissue Engineering

**DOI:** 10.3390/polym16192797

**Published:** 2024-10-02

**Authors:** Angelika Zaszczyńska, Konrad Zabielski, Arkadiusz Gradys, Tomasz Kowalczyk, Paweł Sajkiewicz

**Affiliations:** Institute of Fundamental Technological Research, Polish Academy of Sciences, Pawińskiego 5B, 02-106 Warsaw, Poland; azasz@ippt.pan.pl (A.Z.); kzab@ippt.pan.pl (K.Z.); argrad@ippt.pan.pl (A.G.); psajk@ippt.pan.pl (P.S.)

**Keywords:** piezoelectricity, scaffolds, smart scaffolds, PVDF, PLLA, PVDF-TRFE, collagen, keratin, tissue engineering, bone tissue engineering, smart medicine, regenerative medicine

## Abstract

Bone repair and regeneration require physiological cues, including mechanical, electrical, and biochemical activity. Many biomaterials have been investigated as bioactive scaffolds with excellent electrical properties. Amongst biomaterials, piezoelectric materials (PMs) are gaining attention in biomedicine, power harvesting, biomedical devices, and structural health monitoring. PMs have unique properties, such as the ability to affect physiological movements and deliver electrical stimuli to damaged bone or cells without an external power source. The crucial bone property is its piezoelectricity. Bones can generate electrical charges and potential in response to mechanical stimuli, as they influence bone growth and regeneration. Piezoelectric materials respond to human microenvironment stimuli and are an important factor in bone regeneration and repair. This manuscript is an overview of the fundamentals of the materials generating the piezoelectric effect and their influence on bone repair and regeneration. This paper focuses on the state of the art of piezoelectric materials, such as polymers, ceramics, and composites, and their application in bone tissue engineering. We present important information from the point of view of bone tissue engineering. We highlight promising upcoming approaches and new generations of piezoelectric materials.

## 1. Introduction

Piezoelectric materials (PMs) are smart materials (SMs) that can generate electrical signals in response to applied factors. In 1880, Pierre and Jacques Curie discovered piezoelectricity through deformation, resulting in a shift in ions or charges. This phenomenon includes electricity generation and electric polarization changes [1].

PMs dedicated to bone tissue engineering (BTE) have been gaining attention since 2016 [2]. Generally, the biological properties of the selected material depend on its ability to mimic human tissues [3], and in piezoelectric materials, the electric stimuli are crucial [4]. External electrical stimulation, electrical fields, and bioelectrical signals are important factors due to their contribution to bone repair. PMs can deliver electrical signals without external stimuli and increase the bone’s ability to regenerate [5,6,7,8]. Also, these specific types of materials are known for the converse piezoelectric effect. Thus, electromechanical behavior can be managed by physiological electrical changes as mechanical signals appear. There is an increasing amount of research on the use of piezoelectric nanomaterials in bone tissue engineering. Especially since 2016, the number of publications has increased dramatically. The state of the-art shows the need for novel materials to restore bone fractures [9]. 

**Figure 1:**
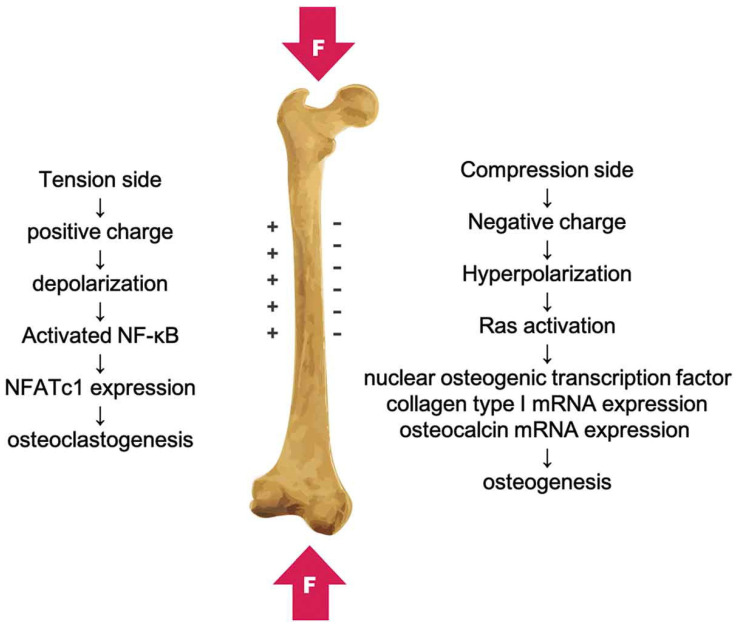
The piezoelectricity in bone caused by mechanical deformation with positive and negative charges. Reprinted with permission from Ref. [14]. Copyright 2019, Taylor and Francis Group.

Piezoelectric, ferroelectric, and pyroelectric signals are the bioelectrical signals that occur in a naturally living bone. It has been proven that these factors influence the healing, remodeling, and growth of bone structures [10,11]. Bioelectricity in the living bone was found to be connected with the non-centrosymmetry and piezoelectricity of collagen molecules [12]. The electric field in bones supports cell proliferation and growth. It has been noted that the polarity of the piezoelectric charges depends on the direction of bone deformation or mechanical stress, with positive and negative piezoelectric charges (Figure 1). The piezoelectric constant (d_33_) of bone has been found to range from 0.7 to 2.3 pC/N^−1^ [13].

The piezoelectric potential of human bone during walking is around 300 μV [15]. Information about the piezoelectric and dielectric properties of bone (trabecular and cortical) and articular cartilage is shown in Table 1.

PMs generate electrical charges on the surface under an external stimulus, similarly to bone. It was reported that polarized surfaces can improve the osteogenic properties of natural bone [19]. Also, piezoelectric implants promote bone tissue repair [20]. Implementing the piezoelectric scaffold into the bone defect causes electrical stimulation through physiological loads [21]. During activation of the cell membrane, calcium channels are open, providing hyperpolarization. 

**Figure 2:**
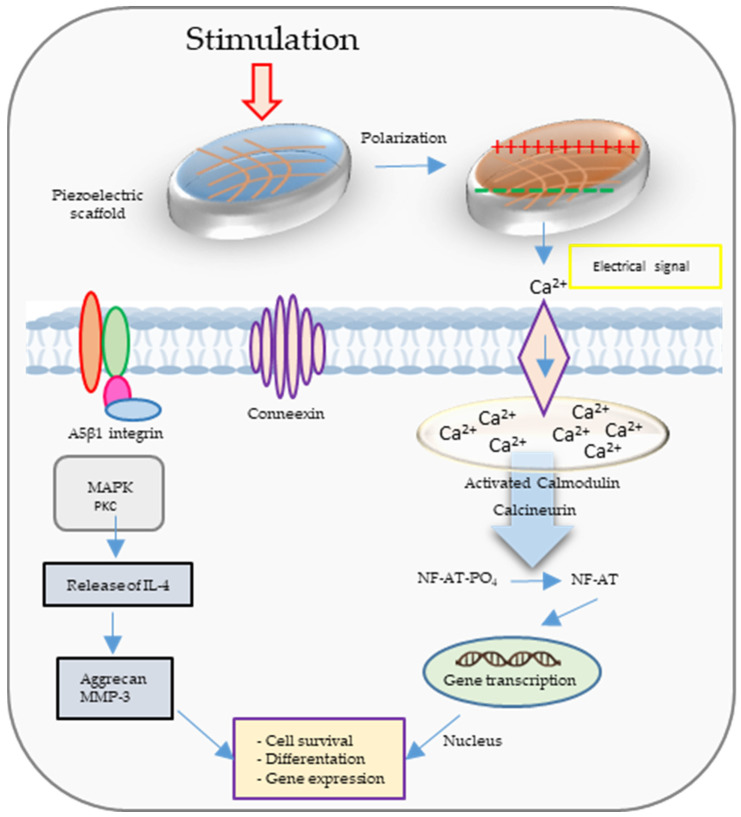
The calcium ion (Ca^2+^) signal transduction pathway and several additional signaling pathways are activated through electrical and mechanical stimulation.

Figure 2 illustrates the calcium ion (Ca^2+^) signal transduction pathway and several additional signaling pathways triggered by both electrical and mechanical stimulation. When mechanical forces are applied to a piezoelectric scaffold, they generate electrical signals that, in turn, activate voltage-gated Ca^2+^ channels. This leads to an increase in intracellular Ca^2+^ concentration, subsequently initiating the activation of calcium-modulated protein (calmodulin). Calmodulin, in turn, triggers the activation of calcineurin, a calcium- and calmodulin-dependent serine/threonine protein phosphatase. Once activated, calcineurin dephosphorylates NF-AT (nuclear factor of activated T-cells), causing it to translocate to the nucleus, where it collaborates with other associated proteins as transcription factors. Furthermore, mechanical stimulation itself can activate mechanoreceptors within the cell membrane, ultimately initiating the activation of protein kinase C (PKC) and mitogen-activated protein kinase (MAPK) signaling cascades. These cascades contribute to the synthesis of proteoglycan and the inhibition of interleukin-1 (IL-1), which is responsible for the degradation of proteoglycan [22].

The role of intracellular Ca^2+^ ions is significant in cellular proliferation [23]. Hence, it can be deduced that the use of piezoelectric biocompatible scaffolds may lead to early restoration of damaged tissue compared to their non-piezoelectric counterparts [24]. Liu et al. [25] examined the osseointegration of various implants by implanting positively polarized bismuth ferrite perovskites membrane–strontium titanate, a negatively polarized strontium titanate membrane–strontium titanate implant, and a non-polarized strontium titanate implant into rat femurs. At the interface of the oppositely charged strontium titanate and bismuth ferrite perovskites implant (+75 mV) and the electronegative bone defect surface (−52 to −87 mV), an inherent electric field was formed [26]. Consequently, this electric field promoted greater osseointegration compared to the strontium titanate and the bismuth ferrite perovskites implant and non-polarized strontium titanate surfaces.

Given the aforementioned context, this review provides an overview of the fundamental concepts, origins, and implications of piezoelectricity in natural bone. Furthermore, it explores the potential of different piezoelectric bioceramics and biopolymers to emulate the electromechanical characteristics observed in living bone. The discussion also addresses the challenges associated with processing and achieving the desired combination of piezoresponse properties, along with potential solutions. Additionally, an objective examination of the beneficial effects of polarized piezoelectric substrates on enhancing biocompatibility is included.

## 2. The Role of Piezoelectricity in Bone Tissue Engineering

**Figure 3:**
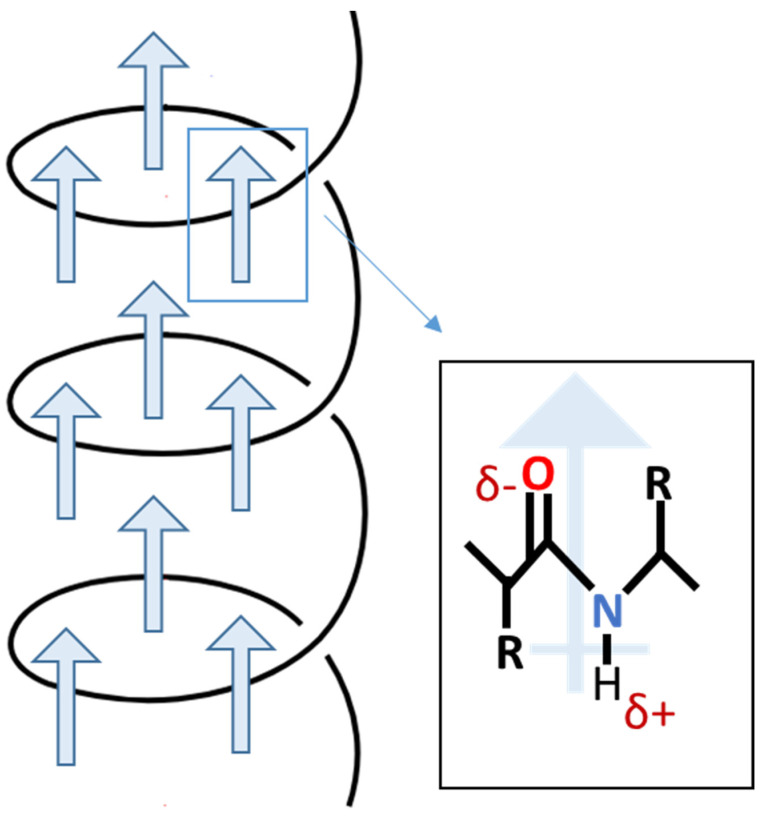
Scheme of permanent polarization in α-helix.

In 1940, Martin [27,28] observed the initial manifestation of biological piezoelectricity. This occurred during the discovery of electric potentials emanating from compressed wool enclosed in shellac between two brass plates. Mammalian hair, wool, horn, and hoof primarily consist of α-keratin, characterized by a spiral α-helix structure [29]. The piezoelectric properties of these tissues stem from the orderly arrangement of α-helices, promoting inherent polarization [30]. The α-helix, a right-handed coil, achieves stability through hydrogen bonds between the amine and carbonyl groups. As depicted in Figure 3, the helical structure aligns amino acid dipoles, resulting in a substantial and enduring polarization [31].

The piezoelectric properties of bone were first reported by Yasuda in 1954 [32]. Subsequently, Yasuda and Fukada [33] conducted experiments on boiled bone and determined that the piezoelectric response was not attributed to living cells. They concluded that the application of shear on collagen within bone was responsible for its piezoelectric behavior. Bone is a composite material comprising densely packed collagen fibrils aligned with hydroxyapatite particles [34]. Collagen, the most abundant protein in mammals, possesses a triple helix structure known as the “triple helix” [35]. 

In 1892, Julius Wolff [36] proposed that bone adapts its structure in response to mechanical stress, known as “Wolff’s Law”. This principle is evidenced by the denser bone found in the arms of tennis players who hold rackets and the bone loss observed in astronauts. The discovery of piezoresponse in dry bone [32,37] led to the suggestion that piezoelectricity could explain the process of bone growth and resorption in response to stress. One of the pioneers in investigating the biological effects of piezoelectricity, Bassett [38], observed that periodically deformed chick embryonic tibiae produced large periosteal chondroid masses after seven days, unlike undeformed samples. He described Wolff’s Law as a negative feedback loop: the applied load on bone induces strain in less dense regions, while denser and stiffer regions remain unstrained. The strain is converted into an electric field that aligns macromolecules and ions in the extracellular matrix, stimulating bone remodeling by cells until the signal is deactivated.

As piezoelectric measurements expanded to include wet bone [39] and wet collagen [40], which represent physiological conditions, discrepancies were observed in the amplitude and behavior of stress-generated potentials between wet and dry samples, and it was discovered that the induced electric potential and the relaxation time are dependent on the induced potential, which is significantly higher in wet samples. Various hypotheses were proposed to explain these inconsistencies, including the p–n junction characteristic of apatite–collagen [41], which suggested that bone exhibited piezoelectric behavior in one direction and piezoresistive behavior in another. However, a widely accepted notion suggested that while piezoelectricity accounted for stress-induced potentials in dry bone, the mechanism responsible for wet bone was the streaming potential [42,43]. According to the streaming potential theory, the stress-generated potential in wet bone was a result of the flow of ion-containing interstitial fluid through the bone under pressure. This theory gained prominence after Pienkowski et al. [44] reported that the conductivity of the saturating fluid significantly influenced the amplitude and polarity of the generated potentials, and the longer relaxation time was attributed to the fluid’s viscosity.

A more recent theory of mechanosensation in bone proposes that applied stress on bone is translated into biochemical signals through the flow of interstitial fluid into the canaliculi–lacunae space, which supplies bone cells with nutrients and conveys shear stress to cells [45]. Despite these new theories, the debate continues regarding the exclusion of piezoelectricity entirely from mechanosensation.

(1) $$V=\frac{\zeta P\kappa }{4\pi \sigma \eta }$$

where ζ, P, κ, σ, and η are the zeta potential, the pressure on the bone, the dielectric permittivity, the conductivity, and the viscosity of the interstitial fluid, respectively.

## 3. Overview of Piezoelectric Materials for Bone Tissue Engineering

**Figure 4:**
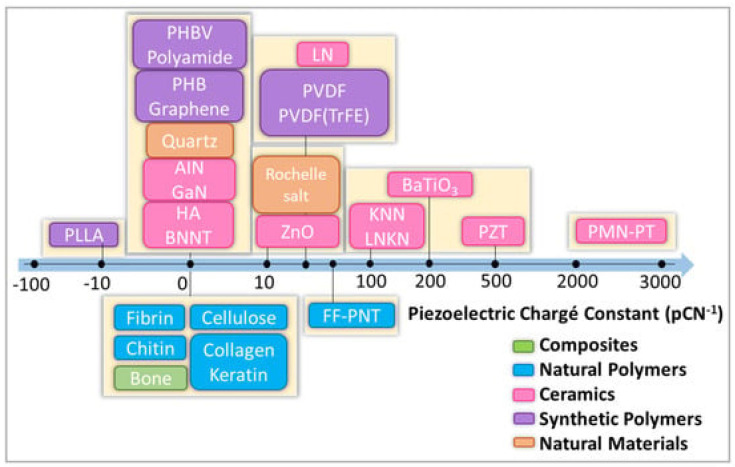
Scheme with classification of selected natural and synthetic piezoelectric materials. HA: hydroxyapatite. Zinc oxide (ZnO), boron nitride (BN), gallium nitride (GaN), poly(vinylidene fluoride-trifluoro ethylene) P(VDF-TrFE), poly(vinylidene fluoride) (PVDF), poly(l-lactic acid) (PLLA), polyhydroxybutyrate (PHB). Reprinted from ref. [46].

Piezoelectric polymers can be divided into natural and synthetic. Figure 4 shows selected piezoelectric materials along with the piezoelectric coefficient (d_33_). 

### 3.1. Natural Polymers

Among the natural polymers are two groups important from the point of view of piezoelectricity: polysaccharides and proteins. Piezoelectric polysaccharides are, e.g., chitosan and cellulose, and piezoelectric proteins are, e.g., keratin, collagen, and fibrin. Table 2 shows piezoelectric coefficients in various types of natural materials.

#### 3.1.1. Chitosan

**Figure 5:**
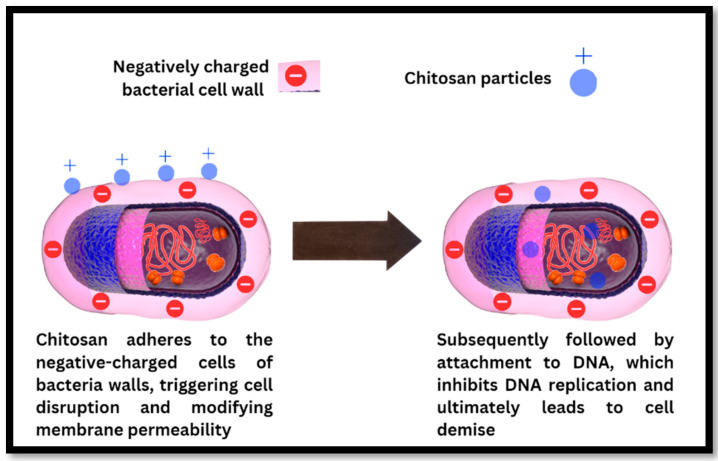
The antibacterial mechanism of chitosan particles involves disrupting the bacterial cell wall, binding to bacterial DNA, and inhibiting DNA replication, ultimately resulting in bacterial cell death. Reprinted from ref. [57].

Chitosan, a naturally occurring polysaccharide polymer with piezoelectric properties, was examined in a comprehensive review by Martino [54]. Studies have shown that polymer scaffolds derived from chitosan possess a range of beneficial features suitable for orthopedic implants, including osteoconductivity, porosity, ease of shaping, antibacterial properties, and minimal foreign body response. Preliminary investigations have documented the piezoelectric sensitivity of chitosan [55], and the current study presents the development of a vibration sensor leveraging chitosan’s piezoelectricity, with the maximum d_33_ coefficient of chitosan amounting to 18.4 pCN^−1^ [47,56]. Chitosan, while regenerating bone defects, may also have antibacterial properties (see Figure 5).

#### 3.1.2. Cellulose

Cellulose, an inherent polysaccharide polymer with piezoelectric properties, emerges as a promising option for bone tissue applications owing to its exceptional biocompatibility and robust mechanical strength. In a study by Zaborowska [58], it was found that employing a microporous cellulose scaffold led to a considerably greater proliferation of MC3T3-E1 osteoblast cells compared to the nanoporous scaffold. Research studies have provided evidence of cellulose’s capacity to enhance cellular adhesion, specifically for chondrocytes, osteocytes, endothelial cells, and smooth muscle cells [59]. As a result, cellulose is regarded as a suitable piezoelectric material for applications in both bone and cartilage tissue engineering [60].

#### 3.1.3. Keratin

Keratin has been utilized to create diverse scaffold architectures suitable for tissue engineering applications. These include hydrogels [61], films [62], sponges [63], microcapsules, and dense materials [64]. Various fabrication techniques, such as solvent casting, lyophilization, electrospinning, and 3D printing, have been employed to produce these keratin-based structures [65]. Furthermore, keratin’s cell recognition domains have been observed to facilitate the attachment of different cell types, including osteoblasts [66].

#### 3.1.4. Collagen

The application of collagen scaffolds in bone healing has been previously reported [67]. Additionally, the effectiveness of collagen–hydroxyapatite piezoelectric composite scaffolds in promoting cellular growth and facilitating bone healing has been demonstrated [68]. Moreover, collagen–calcium phosphate composite scaffolds have been explored for cartilage tissue engineering. Studies involving these scaffolds have shown an average filling ratio of the defect area with newly formed cartilage tissue at weeks eight and twenty to be approximately 81% and 96%, respectively [69]. Nonetheless, it is important to acknowledge certain limitations of these scaffolds, including low mechanical stiffness, rapid degradation, and potential toxicity associated with the use of crosslinking agents. Collagen under stress reduces the hydraulic permeability and increases the stiffness [70], which can be improved through electrical stimulation [71]. Table 3 summarizes information about natural piezoelectric materials and their applications in bone tissue engineering (BTE).

### 3.2. Synthetic Polymers

Piezoelectric synthetic polymers have unique electrical properties that can be used to stimulate bone tissue cells. Thanks to the ability to convert mechanical energy into electrical signals, these polymers are used in the creation of tissue scaffolds that not only mechanically support bone regeneration but also provide electrical stimulation that mimics the natural conditions of the cellular microenvironment [81,82]. The piezoelectric coefficient data for different synthetic piezoelectric materials are shown in Table 4.

#### 3.2.1. Polyvinylidene Fluoride (PVDF)

**Figure 6:**
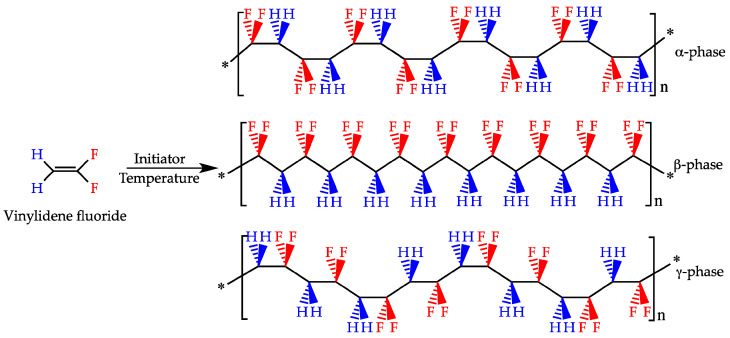
Polymerization of the vinylidene flyuoride monomer with various PVDF chain conformations of α-phase (non-polar) and β- and γ-phases (polar). Reprinted From Ref. [91].

PVDF is a water-insoluble polymer that has gained a lot of attention in a broad range of fields due to its electroactive properties as well as its high chemical and thermal stability. The crystalline phase of PVDF can include five different polymorphs (i.e., α, β, γ, δ, ε) (Figure 6), and their presence is dictated mostly by processing method conditions [86]. PVDF usually crystallizes in the form of α phase due to its thermodynamic stability. However, β and γ phases are very often desired because of their electroactive properties, such as piezoelectricity, pyroelectricity, and ferroelectricity [87,88]. These properties are related to the conformation of their chains, where the γ phase shows a worse piezoelectric effect than the β phase due to a gauche bond existing in every fourth repeating unit [89]. It has been known for some time that β-PVDF induces cellular proliferation and differentiation, especially under dynamic conditions, which is one of the reasons why PVDF is considered a promising material for BTE [90]. This section focuses on advances made in the field of PVDF for application in BTE, and it reviews articles published mainly since 2018 to date, acting as a follow-up to the review by Tandon et al. [2].

**Figure 7:**
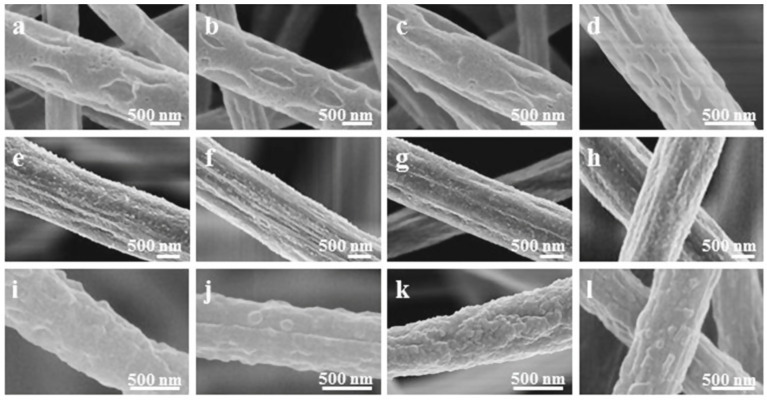
SEM microstructures with PVDF electrospun fibers’ morphology with various applied voltages during the electrospinning process; (**a**,**e**,**i**)—6 kV; (**b**,**f**,**j**)—12 kV; (**c**,**g**,**k**)—18 kV; (**d**,**h**,**l**)—24 kV. Reprinted with permission from ref. [96]. Copyright 2019, IOP Publishing Group.

Recently, great advances have been made to address one of the main limiting factors to the widespread use of PVDF in BTE, which is its hydrophobic profile. Kitsara et al. used oxygen plasma treatment for electrospun and solvent-cast scaffolds and proved that the enhanced hydrophilic profile of PVDF can be maintained even up to two years, resulting in better cell spreading and interaction with the scaffold [92]. Hydrophilicity was also improved through spin coating of PVDF onto the TiO_2_ nanotube surface of titanium, which halved the contact angle after PVDF polarization and promoted mineralization, but maintaining these properties long-term is questionable due to the loss of negative charge [93]. A different approach to address poor cell–scaffold interaction due to the hydrophilicity of PVDF was proposed, in which PVDF membranes were coated with elastin-like recombinants containing l-arginyl-glycyl-l-aspartic acid sequence (RGD) motifs, and it improved MSCs’ adhesion and proliferation [94]. As mentioned previously, the β phase is the most desirable for BTE, and during the electrospinning process, a fraction of this phase can be increased by adjusting the accelerating voltage towards higher values [95]. Therefore, to obtain a significant amount of β phase, the piezoelectricity and the molecular weight can be increased. The surface is shown in Figure 7.

Recently, Lee et al. produced a three-dimensional cotton-like scaffold by changing the relative humidity and compared different post-treatment methods (i.e., heating, cooling, quenching) to increase the β-PVDF content, from which cooling showed the best results [97]. A comparison of 2D and 3D structures was conducted for solvent casting methods at different temperatures, and although the room temperature process resulted in higher amounts of β-PVDF and crystallinity, their hydrophobicity was greater compared to high-temperature solvent casting [98]. Surprisingly, their results also showed that 2D films promote cell proliferation better than 3D scaffolds. Mirzaei et al. came to different conclusions when comparing 2D films with 3D electrospun fibers and showed that the latter is better at improving the osteogenic differentiation of induced pluripotent stem cells (iPSCs) [99]. Different studies tested a freeze-extraction method to produce PVDF membranes with controlled microporosity. However, their control samples (glass slides) showed better proliferation of MSCs than the obtained membranes [100]. Szewczyk et al. showed that the surface potential of PVDF electrospun fibers has a significant effect on the mineralization rate and cell attachment, and it can be controlled through polarity during the electrospinning process [101]. Moreover, it was shown that Kelvin probe force microscopy is a viable method to measure the surface potential of electrospun fibers. 

Studies on PVDF copolymers and composites have been focused mainly on difficulties with the fabrication of scaffolds with proper topography and spatial morphology and maintaining sufficient piezoelectric properties. For example, poly(vinylidene fluoride-hexafluoropropylene) (PVDF-HFP) copolymer was used to produce a termite-like scaffold using the wet electrospinning method, which resulted in improved electric output and promoted NIH 3T3 cell migration and attachment in comparison to regular electrospun fibers [102]. On the other hand, Zhou et al. fabricated electrochemically polypyrrole (PPy) nanocones on the surface of the PVDF membrane, which enhanced bone marrow mesenchymal stem cells’ (BMSCs) spreading, adhesion, and osteogenic differentiation [103]. Cell stimulation can be realized through the pulsed electromagnetic field (PEMF) method, and it was shown that the presence of conductive polymer polyaniline (PANI) in the electrospun PVDF fibers not only improves biocompatibility and osteoinductivity of copolymer but also enhances the effect that PEMF has on dental pulp stem cells’ (DPSCs) osteogenic differentiation [104].

#### 3.2.2. Poly(Vinylidene Fluoride-Trifluoroethylene) (PVDF-TRFE)

**Figure 8:**
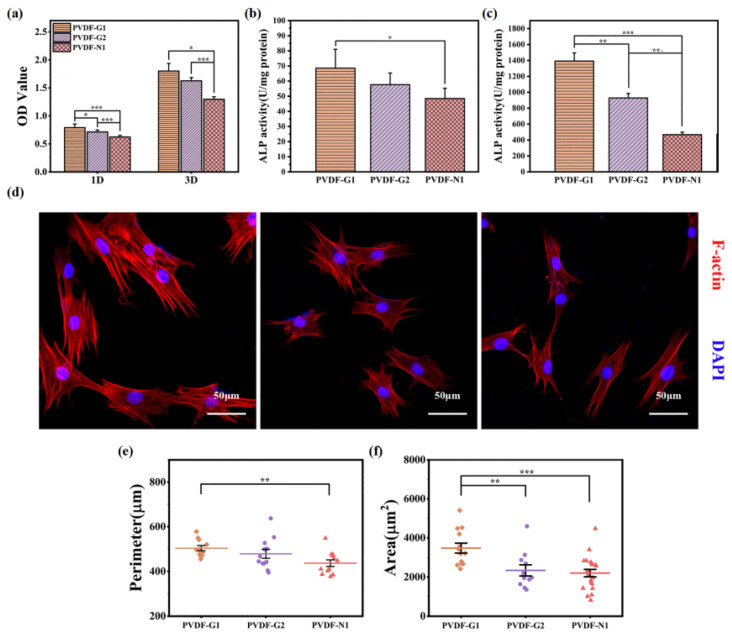
Selected parameters of scaffolds. (**a**) The adhesion (day 1) proliferation (day 3) behaviors of MSCs on PVDF-TrFE; (**b**,**c**) ALP activity of MSCs using PVDF-TrFE after 7 and 14 days. (**d**) Microstructures of CLSM of MSCs on PVDF-TrFE after 3 days. (**e**,**f**) Cytomorphometric tests of MSCs on PVDF-TrFE for 3 days. Reproduced with permission from the publisher, reference [112].

This polymer is a copolymer composed of vinylidene fluoride (VDF) and trifluoroethylene (TrFE). Among the various polymers, this particular copolymer has demonstrated the highest piezoelectric coefficient (30 pCN-1) [33,105]. Research indicates that the copolymer exhibits cytocompatibility and exerts a positive influence on cell adhesion and proliferation. Notably, it possesses regenerative capabilities for various tissue types, including bone, skin, cartilage, and tendons [106,107]. Electrospun nanofibrous scaffolds based on the PVDF-TrFE copolymer have shown remarkable efficiency in the regeneration of bone, cartilage, and others [108]. In addition, PVDF and PVDF-TrFE have been blended with natural polymers, such as starch or cellulose, to develop suitable scaffold structures for tissue repair and regeneration, especially in the field of bone tissue engineering. Blending starch or cellulose produces a porous structure that supports tissue growth [109]. Scientists [110] showed increased bone growth when a thin film of PVDF-TrFE with 10 vol% BaTiO_3_ piezoelectric composite, thermally treated at 1200 °C for 4 h, was implanted into the tibiae of male rabbits for 21 days. The formation of new bone was stimulated through electrical signals generated from the strained piezoelectric membrane during the rabbits’ physical activity. Lopes et al. [111] conducted a comparison between the P(VDF-TrFE) 10 vol% BaTiO_3_ piezoelectric composite and polytetrafluoroethylene (PTFE) to evaluate new bone formation by implanting them in rat calvarial defects for 4 and 8 weeks. Their findings suggest that the piezoelectricity and hydrophilicity of the P(VDF-TrFE)–BaTiO_3_ composite enhance the protein binding affinity, leading to increased osteogenesis. Light microscopy and micro-CT images demonstrated significant hard tissue growth and connective tissue formation at the interface of the implanted piezoelectric membrane in both implants. Scientists [112] have discovered that the responses of bone marrow macrophages (BMDMs) and bone marrow mesenchymal stem cells (BMSCs) to PMMA-coated PVDF-TRFE scaffolds can be controlled by changing certain parameters, such as the surface potential, which in turn enables targeted bone regeneration (see various parameters in Figure 8).

#### 3.2.3. Barium Titanate (BaTiO_3_, BT) as PVDF Dopant

In the literature, one of the most commonly found constituents of PVDF composites is barium titanate (BaTiO_3_, BT), mainly due to its outstanding ferroelectric properties, high dielectric constant, and decent biocompatibility [113]. Recently, a PVDF/BT composite was fabricated for the purpose of testing whether electrical stimulation in the form of a monophasic direct current, a square waveform, and a biphasic waveform has a direct effect on hMSCs’ differentiation. The presented results show that the biphasic waveform does not induce osteogenic differentiation, whereas square wave stimulation does show osteogenic lineage commitment with lower reactive oxygen species (ROS) compared to direct current stimulation [114]. Similar studies explored a composite made from PVDF/BT with a multiwalled carbon nanotube (MWCNT) addition and its combined effect with direct current stimulation of MC3T3-E1 cells, which resulted in a significant improvement in cell proliferation, migration, and osteogenic differentiation in comparison to an unstimulated composite and PVDF control samples [115]. On the other hand, it was shown that PVDF-TrFE/BT membranes combined with MSCs injection are a viable approach for bone repair in the presence of osteoporosis; however, in vivo studies did not result in full bone repair [116]. Freitas et al. showed in their paper the importance of cell sources used with the PVDF-TrFE/BT membrane for bone repair, and only MSCs from bone marrow enhanced bone formation, while MSCs from adipose tissue did not show significant improvement [117]. An in-depth analysis of the biological processes that occur during MSCs osteogenesis induced through a piezoelectric PVDF/BT composite was conducted, and it was noted that the transformation of physical signals into intracellular mechanotransduction is the main driving factor for the improvement of osteogenesis [118]. The homogeneous distribution of BT filler in a polymeric matrix poses a significant challenge, and functionalization with polydopamine has been proposed as a possible approach [119]. Composites obtained through the selective laser sintering (SLS) method with functionalized BT showed not only homogenous distribution, which resulted in an 11% increase in β-PVDF’s presence and more than tripled the voltage output, but also improved mechanical properties of the scaffold. A follow-up study by the same researchers introduced Ag nanoparticles onto the surface of functionalized BT nanoparticles, thus increasing the conductivity and voltage output of a PVDF composite, as well as providing it with antibacterial properties [120]. A similar but different approach was presented recently, in which PVDF was functionalized with polydopamine and later combined with BT to produce a bioactive coating on a bioinert Ti_6_Al_4_V alloy. Functionalization of PVDF increased the fraction of β-PVDF by 91%, and combining it with BT further enhanced β-PVDF’s presence by 96% [121]. Qi et al. produced core–shell BT nanoparticles through functionalization with polydopamine and high-temperature carbonization, which were combined with PVDF and turned into scaffolds through the SLS method [122]. The results show that carbon-coated BT nanoparticles promote β phase formation and increase composite conductivity and its mechanical properties.

#### 3.2.4. Carbon-Based Materials (GO, MXene) as PVDF Dopants

Graphene oxide (GO) is known for its outstanding conductive properties and has been utilized in several studies as an addition to PVDF. The presence of GO in PVDF scaffolds produced through SLS resulted in higher tensile and compressive strength but also enhanced the transformation of PVDF from α to β phase through fluorine and carbonyl group interactions [123]. The same composite system was used for the fabrication of electrospun fibers, and although cell studies showed improvement in iPSCs’ osteogenic differentiation in comparison to pure PVDF, their mechanical properties were significantly worse [123]. Other studies tried to improve electrospun fibers of PVDF/GO composite by blending it with PVA, which increased the tensile strength almost tenfold and more than doubled the tensile strain and modulus [124]. Moreover, this ternary system of PVDF/PVA/GO showed better osteoinductive properties, cell proliferation, and alkaline phosphatase (ALP) activity in comparison to PVDF/GO and PVDF/PVA. Fu et al. used a different approach and incorporated MXenes into the electrospinning solution of PVDF, which improved the mechanical properties, promoted bone regeneration in vivo, increased the β phase content, and also increased the contact angle, making fibers more hydrophobic [125].

#### 3.2.5. Zinc-Based Materials as PVDF Dopants

Zinc-based materials have garnered interest in bone tissue engineering due to their great antibacterial properties. The addition of zinc oxide (ZnO) into electrospun PVDF fibers promoted β phase formation, significantly improved its mechanical properties, and provided the scaffold with antibacterial properties, which were better when piezo-excitation was applied by Li et al. [126]. Their in vitro studies on human osteoblasts resulted in lower cell density after three days without piezo-excitation in comparison to the control group, but with stimulation, cell density was higher for the tested composite. Similar results were obtained by Xi et al., who conducted studies on the same composite in comparable conditions [127]. An interesting approach was recently proposed in which a hybrid system composed of PVDF piezoelectric nanofibers with ZnO nanorods and polycaprolactone (PCL) nanofibers with dexamethasone-loaded chitosan nanoparticles were fabricated using a dual electrospinning method [128]. The obtained results showed that increasing the content of ZnO nanorods simultaneously increases β phase content and that the hybrid scaffold induces osteogenic differentiation of mouse bone marrow stromal stem cells (mBMSCs) and improves their proliferation. On the other hand, Chen et al. used zinc-based metal–organic frameworks (ZIF-8) and, through shear milling and salt leaching methods, produced PVDF/ZIF-8 foams, which were able to generate 10 V outputs without polarization and showed angiogenic, osteogenic, and antibacterial activity [129].

#### 3.2.6. Hydroxyapatite (HA) as PVDF Dopant

One of the most popular groups of materials applied in BTE is calcium phosphates. For example, studies conducted on electrospun PVDF fibers with the addition of hydroxyapatite (HA) showed no cytotoxicity; apatite growth was observed, but the presence of HA slightly decreased the amount of β phase [130]. Other studies used a different approach and first produced electrospun PVDF fibers, which were later treated with plasma, and HA was electrodeposited on their surface [131]. Obtained scaffolds showed a highly hydrophilic character due to plasma treatment. They possessed antibacterial properties, and they promoted MG63 cell proliferation. However, no significant improvement was observed in comparison to reference samples. PVDF coating on the anodized surface of titanium was modified with HA, which decreased the contact angle by 66% for 20% HA content, and enhanced mineralization was observed [132]. Malherbi et al. produced PVDF/β-TCP/HA discs and showed that under oscillating electric fields, the apatite growth rate is increased in SBF in comparison to no stimulation [133]. In other studies, Ca-P-Si-derived material was combined with PVDF, and through the phase separation–hydration method, porous scaffolds were produced that did not show cytotoxicity, had mechanical properties similar to cancellous bone, and promoted HA deposition and osteoblast redifferentiation. However, studies have compared scaffolds with different contents of calcium phosphate silicate (Ca-P-Si) and lack reference to pure PVDF scaffolds [134]. Ma et al. fabricated a PVDF-HFP scaffold through dipping and phase separation methods, which were modified with calcium phosphate (CaP). The resulting scaffolds showed a hydrophilic profile, promoted bone repair in rats, and were capable of generating electrical signals through body movement [135]. PVDF has limited biodegradation in the physiological environment, and to address this issue, PVDF/HA electrospun fibers were produced with the addition of PCL to the electrospinning solution [136]. Although these scaffolds showed cytocompatibility, cell proliferation was worse in comparison to PCL after three days, and only a small increase in ALP activity was observed. Moreover, degradation studies were not included despite being one of the main reasons for PCL addition. In a different approach, polycaprolactone–tricalcium phosphate (PCL-TCP) films were coated in PVDF through a self-polarization technique, which resulted in enhanced osteoinduction through the synergistic effect of polycaprolactone/tricalcium phosphate/poly(vinylidene fluoride) (PCL/TCP/PVDF) piezoelectricity and PEMF stimulation [137]. Alimohammadi et al. used PVDF to improve the bioactivity of sulfonated PEEK and further modified it with HA and carbon nanofibers, which resulted in an improved hydrophilic profile, apatite formation, and MG-63 cell attachment and proliferation [138]. Because the fabrication of PVDF scaffolds with a developed spatial structure is difficult, a UV-curable poly(glycerol azelaic acid)-g-glycidyl methacrylate (PGAz-g-GMA) resin was used and modified with PVDF, HA, and Cloisite Na+ [139]. These scaffolds proved to be mechanically compliant with cancellous bone tissue, the hydrophilic profile was improved due to nanofillers, and the attachment and proliferation of MG-63 cells were observed.

#### 3.2.7. Cobalt Ferrite (CoFe_2_O_4_, CFO) as PVDF Dopant

A few studies have tried to incorporate CFO into PVDF to produce magnetoactive scaffolds. Fernandes et al. used a solvent casting method to develop a porous PVDF/CFO scaffold with the help of a nylon template, which was later removed [140]. The addition of CFO slightly increased the β phase fraction and decreased contact angle, and it showed improved cell proliferation with magnetic stimulation. Other studies have focused on providing magnetoelectric properties for injectable methacrylated gellan gum (GGMA) hydrogel through the incorporation of CFO/PVDF spheres in its structure; however, the obtained scaffold showed worse cell viability in comparison to control samples [141]. Recently, PVDF/CFO spheres were produced using the electrospraying method and introduced into gelatin hydrogel for the purpose of guiding MSCs towards osteogenic differentiation, but magnetic stimulation was shown to have a minimal effect in this regard [94].

#### 3.2.8. Poly(L-Lactic Acid) (PLLA)

PLLA is the second most popular piezoelectric polymer utilized in BTE. One of the main advantages that PLLA has over PVDF is that it is biodegradable; however, similarly to PVDF, it also suffers from hydrophobicity, which hinders scaffold–cell interaction. Moreover, its piezoelectric properties are usually weaker than those of PVDF, which is why it is less commonly found in the literature. Nevertheless, in recent years, several noteworthy advances have been made.

The fused deposition modeling (FDM) method was used to fabricate computer-designed PLLA scaffolds with high porosity, and mechanical testing has shown that the obtained properties are similar to those of trabecular bone [142]. Moreover, scaffolds maintained piezoelectric properties after the 3D printing process, but crystallinity was lower in comparison to the initial filament. Similarly to what has been shown for PVDF, the voltage polarization applied during the electrospinning process has a direct effect on the surface potential and piezoelectricity of PLLA fibers [143]. Positive polarization resulted in better MG-63 cell adhesion and spreading. In a different study, PLLA electrospun fibers were used in pairs with externally controlled ultrasound to stimulate stem cells in vitro and induce bone growth in vivo [144]. The synergistic effect of the scaffold and the stimulation exhibited positive outcomes in both situations. Tai et al. showed that the fiber diameter and heat treatment of electrospun PLLA fibers affect the voltage output. Heat treatment above the glass transition temperature of PLLA simultaneously increases longitudinal and decreases transverse voltage output. They also showed that neurogenic differentiation of stem cells was improved for orthogonal piezoelectricity, whereas osteogenic differentiation was improved for shear piezoelectricity [145]. The novel approach proposed by Lai et al. produced an electrospun PLLA scaffold with extracellular vesicles for the treatment of cartilage defects [146]. Piezoelectric stimulation improved the migration and proliferation of chondrocytes and increased the retention of extracellular vesicles. Liu et al. conducted studies on PLLA scaffolds designed for a cartilage–bone interface in which gradient polarization was applied [147]. This approach resulted in scaffolds with gradient piezoelectric properties in which stronger piezoelectricity guided MSCs towards osteogenic differentiation, whereas weaker piezoelectricity guided them towards chondrogenic differentiation. In a different study by the same first author, the PLLA scaffold was tested in vivo to evaluate the effect of exercise on cartilage regeneration [148]. Although the presence of the scaffold improved cartilage healing in rabbits, the results regarding the impact of exercise are inconclusive. As mentioned previously, the hydrophobicity of PLLA is a significant obstacle towards functional BTE scaffolds, and thus plasma treatment was applied with argon (Ar) and oxygen (O_2_) on electrospun membranes, which had no significant impact on the physicochemical properties of PLLA but increased the surface roughness and decreased the contact angle [149]. 

On the other hand, piezoelectric stimulation has been shown to provide antibacterial properties. Ando et al. produced PLLA yarns and tested them against Staphylococcus Aureus, which proved their antibacterial properties during mechanical deformation [150]. However, the mechanism through which bacteria are killed was not fully explained. Their studies were later expanded by Gazevoda et al., who obtained similar results on nanotextured films but showed that the impact of reactive oxygen species and pH changes is insignificant and bactericidal properties are attributed to the piezo stimulation [151].

#### 3.2.9. Barium Titanate (BT) as PLLA Dopant

Antibacterial and anti-inflammatory properties were obtained for PLLA solvent-cast membranes through the addition of electrospun BT nanofibers co-doped with Ca/Mn [152]. PLLA scaffolds showed increased electroactivity, osteogenesis, and mineral deposition in the presence of nanofiller. Other authors have addressed the issue of dielectric differences between ceramic BT and PLLA by introducing graphene oxide into this composite [153]. They produced scaffolds using the SLS method and showed that the addition of GO improved the output voltage and the current and promoted cell proliferation and differentiation, especially when stimulated with ultrasounds. The orientation of PLLA/BT electrospun fibers has been shown to affect the differentiation of BMSCs and the electrical properties of the scaffold [154]. Randomly oriented PLLA fibers generally showed better properties by promoting osteogenic differentiation and higher dielectric permittivity, which was further improved by the presence of BT, which simultaneously decreased the contact angle and increased the surface roughness. In a recent study, the effects of BT morphology (i.e., nanoparticles, nanosheets, nanotextured rods, microblocks) incorporated into PLLA film were evaluated. The results showed that high-aspect-ratio structures of the filler are preferable for obtaining highly crystalline PLLA, which translated into better cellular proliferation [155].

#### 3.2.10. Hydroxyapatite (HA) as PLLA Dopant

**Figure 9:**
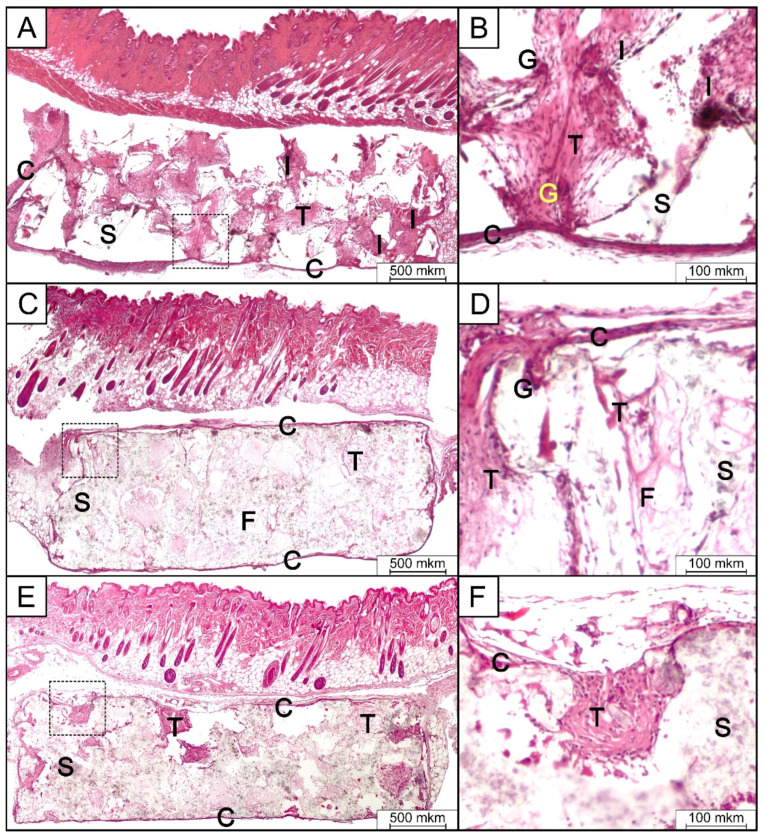
Micrographs of H&E-stained tissue sections from the subcutaneous implantation area in group 1 (PLA) (**A**,**B**), group 2 (PLA/HA 15%) (**C**,**D**), and group 3 (PLA/HA 20%) (**E**,**F**) after 14 days of implantation. S—scaffold; C,T—connective tissue; F—collagen fibers; I—inflammatory infiltrates; G—giant multinucleated cell. (Reproduced with the permission from the publisher, reference [162]).

In the literature, there is an abundance of reports regarding PLLA/HA composites; however, the majority of these studies focus either on piezoelectric properties for non-biomedical applications (e.g., energy harvesting [156,157]) or strictly on the aspect of cellular scaffolds for BTE, excluding the piezoelectric effect [158,159,160]. We have failed to find a study that evaluates both of these for the same PLLA/HA platform. HA dopants for the PLLA were tested in vivo to produce plates for the fixation of bone fractures [161]. Subcutaneous implantation of PLA/HA [162] scaffolds in mice showed good tolerance of all scaffolds and tissue growth. Samples containing HA showed a lower inflammatory response after 14 days of implantation compared to PLA samples (see Figure 9).

#### 3.2.11. Carbon-Based Materials (GO, MXene) as PLLA Dopants

A different study incorporated graphene oxide (rGO) into PLLA and modified produced electrospun fibers with perylene-3,4,9,10-tetracarboxylic dianhydride (PDA) to increase the attachment of chondrocytes [163]. The results showed that not only piezoelectricity but also the degradation profile have a direct effect on clonal mouse embryonic cell line (ATDC5) cell differentiation. Pariy et al. also modified PLLA electrospun fibers with rGO but focused their studies on its effect on piezoelectricity, which was increased in and out of plane 2.3 and 15.4 times, respectively [164]. Moreover, they showed that the piezoresponse of the PLLA composite is dependent on the molecular structure. Other authors showed that coating PLLA electrospun fibers with PANI and carbon nanotubes (CNTs) deteriorates the mechanical properties but lowers the electrical resistance and increases the voltage output [165]. The addition of conductive components improved human-bone-marrow-derived mesenchymal stem cells’ (hBMMSCs) proliferation and osteogenic differentiation only when stimulated through ultrasonication. Ramasamy et al. decided to go a step further and incorporate into the PLLA fibers two pluronic F-127 (PL) functionalized fillers, with one improving conductivity (PL-MWCNT’s) and the other improving piezoelectricity (PL-BN) [166]. This complex system resulted in increased mechanical properties and an improvement in wettability and electroactivity, and it promoted MC3T3-E1 cell adhesion and proliferation. Table 5 summarizes information about synthetic piezoelectric materials and their applications in BTE.

### 3.3. Other Piezoelectric Polymeric Materials 

As previously described, PVDF and PLLA are the most commonly found piezoelectric polymers in the literature for use in BTE. However, two alternatives have been proposed to date that might be suitable for scaffold design. This section will briefly characterize them by showing their advantages and disadvantages, but it will also mention the most significant advances.

Poly(3-hydroxybutyrate) (PHB) is a part of the polyhydroxyalkanoate family, biodegradable bacterial polyesters that have been found to be used in different branches of medicine [185,186,187]. PHB is considered to be highly biocompatible due to its degradation product being a natural metabolite, and it does not induce inflammatory reactions. Moreover, it possesses piezoelectric properties, which are attributed to the presence of a polar oxygen group linked with an asymmetric carbon [188]. However, PHB has a low degradation rate, it may contain contaminants causing adverse effects, it is hydrophobic and brittle, and its piezoelectric coefficient is significantly lower than that of PVDF and PLLA [189]. Nevertheless, several studies have explored the viability of PHB, its blends, and composites, mostly in the form of electrospun fibers [190,191] but also as 3D-printed scaffolds [6,192], hot-pressed films [193], solvent-casted films [194,195], and lyophilized films [196].

Polyamide-11 (PA-11) is a synthetic polymer with better piezoelectric and mechanical properties than PHB, but its non-biodegradable character limits its application in BTE. PA-11 can crystallize into five different phases, from which the polar pseudo-hexagonal γ phase exhibits the highest piezoelectricity [197]. Despite the popularity of polyamides in the biomedical field, very few researchers have decided to study PA-11 for BTE. In 2007, Wang et al. produced a PA/HA composite scaffold through the phase inversion method and showed that the obtained structures are characterized by exceptional mechanical properties, promote MSCs’ osteoblastic differentiation, and induce bone formation in vivo [198]. However, the study did not consider the piezoelectric aspect of polyamide. A more recent study produced piezoelectric PA-11 nanoparticles for ultrasound stimulation of stem cells towards osteogenic differentiation [197]. In their paper, the authors stated that synthesized particles are endocytosed by stem cells and exhibit great potential in regulating their fate under stimulation. Despite the fact that PA-11 is not biodegradable, its potential is underappreciated, and it is expected that in the upcoming years, more researchers will include it in their studies either as a standalone scaffold or as a bioactive coating for inert implants.

## 4. Stimulation and Biological Properties of Piezoelectric Materials

The introduction of piezoelectric material stimulation into tissue engineering opens up perspectives for more effective and precise therapies in the healing and regeneration processes of bone tissue. Determining how mechanical stimulation of these materials can affect cells and tissues, with a particular emphasis on bone tissue regeneration processes, is crucial for improving the quality of treatment [2].

Multiple stimulation factors can activate the piezoelectric properties of scaffolds. It was reported that cells, through adhesion to the surface of the scaffold, can exert enough strain to cause polarization and an electrical response [135]. Moreover, the surface polarity of piezoelectric material can affect the conformation of adsorbed proteins from the extracellular matrix (ECM), thus possibly improving adhesion and osteogenic differentiation [123].

Optimizing the piezoelectric effect of biomaterials for biomedical applications requires careful adjustment of many material and process parameters that affect their mechanical and electrical properties [2]. In the context of tissue regeneration, such as bone, piezoelectric biomaterials can convert mechanical stimuli into electrical signals that support natural biological processes. The key is to optimize the piezoelectric effect, which starts with the selection of the appropriate material depending on the application. More specifically, piezoelectric composites that combine the flexibility of polymers with the higher piezoelectricity of inorganic materials can be an interesting choice. The structure of the piezoelectric material also plays a role [199]. The orientation of the fibers (e.g., [46]) can increase their ability to generate electrical signals. Appropriate mechanical properties are also important so that the material has mechanical properties similar to bone tissue, which prevents damage to the newly formed tissue. The introduction of appropriate additives [200] and the adjustment of the biodegradation rate [201] also play a key role. Only through a comprehensive approach is it possible to obtain biomaterials that will effectively support regenerative processes while offering long-term stability and biocompatibility.

Mechanical stimulation is the simplest stimulus that also naturally occurs during movement and can be easily applied to a scaffold. Piezoelectric scaffolds induce better attachment, proliferation, and differentiation of cells under dynamic culturing conditions [90,98,103]. On the other hand, in vivo studies have shown that piezoelectric scaffolds perform significantly better during regular exercises, which improves overall chondrogenic differentiation and cartilage formation in rabbits [148]. The combination of electrical stimulation (ES) and conductive polymers, which strengthen the local electrical field, has been known to improve osteogenic activity. This synergistic effect favors mineral nucleation and protein absorption, accelerates the transport of Ca^2+^ ions into the cells, and upregulates the mitochondrial activity [202,203,204,205,206]. Bhaskar et al. constructed an in-house 12-well plate device to study the effects of electrical stimulation on cell culture and showed that ES promotes cell migration, spreading, and attachment on the piezoelectric PVDF/BT/MWCNT scaffold in comparison to a lack of stimulation [115]. The effect of the waveform on osteogenesis was recently evaluated by Panda et al. [114]. It was found that the direct current causes higher levels of intracellular ROS and induces early osteogenesis in MSCs, whereas the square wave produces lower levels of ROS and affects late osteogenesis. On the other hand, one study showed that the PVDF scaffold does not induce apatite nucleation in simulated body fluid (SBF) with and without ES, but PVDF/HA composite does [133,186]. It can be concluded that electrical stimulation can positively affect bone regeneration [23,207] and control the movement of tested cells [208]. 

**Figure 10:**
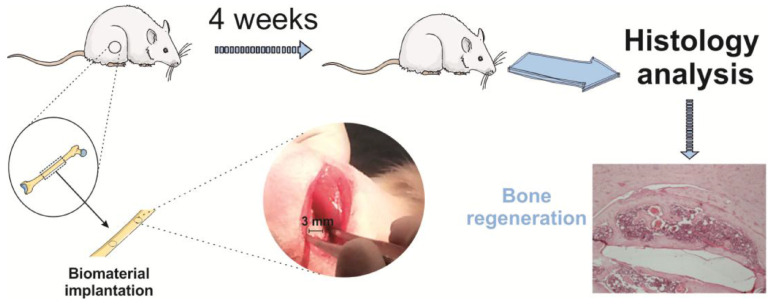
A visual depiction of the biomaterial implantation surgical protocol and a typical image of Hematoxylin and Eosin (H&E) staining showing the healing defects four weeks post-implantation on a poled β-PVDF film. Reproduced with permission [170].

Negatively charged PVDF scaffolds [167] promoted the osteogenic differentiation of human-adipose-derived stromal cells. Combining mechanical stimuli with biochemical stimuli successfully replicates the biomimetic microenvironment of the human body. These findings were validated by in vivo studies in which PVDF films were tested for their osteogenic properties in Wistar rats by analyzing new bone formation in a bone defect model [170]. After four weeks, significantly more defect closure and bone remodeling were observed (Figure 10). In this case, the mechanical stimuli were provided by the movements of the rats.

The electromagnetic field (EMF) is another stimulus that can be considered in combination with a piezoelectric scaffold to improve overall performance. Although there are reports stating that it can have a negative impact on different physiological processes, the extremely low-frequency EMF shows therapeutic potential in bone tissue treatment [209,210]. Mirzaei et al. showed that the dental pulp stem cells seeded on piezoelectric scaffolds have better attachment and viability, and protein adsorption is increased, when EMF is applied [104,211].

In the literature, ultrasound stimulation has shown promising results in bone and cartilage tissue engineering. Chen et al. conducted in vivo studies by implanting PVDF/ZIF-8 foam into femur defects of rats [129]. The piezoelectric component was activated through ultrasonic stimulation for 20 min at 1000 Hz three times a day. This resulted in improved angiogenesis and osteogenesis, as well as upregulated ions transported into the cells. Cell adhesion, proliferation, and differentiation on PVDF composite scaffolds were also significantly improved when ultrasounds were applied [119,120,123]. Similar results were also obtained for piezoelectric PLLA scaffolds [155]. Das et al. conducted extensive in vitro and in vivo studies and found that cellular osteogenesis, as well as bone defect healing, is best when both the piezoelectric scaffold and ultrasound (US) are applied synergistically [144]. The piezoelectric material can be used as a coating for metallic bone implants and, in a similar manner, also improve osteogenesis with the help of US [212]. In subsequent work, scientists investigated PVDF scaffolds in vitro with [213] and without [46] ultrasound. It has been shown that appropriate ultrasound power can increase cell proliferation; in this case, it was 80 mW.

In the context of preclinical evaluation, it was observed that polarized PVDF exhibited significantly higher osteogenic efficacy compared to a non-polarized polymer substrate when implanted into the interosseous membrane of the rat tibia for six weeks. It is indicated that enhanced bone regeneration can be attributed to piezoelectric signals generated by polarized surfaces. Polarized PVDF films and fibers facilitate enhanced bone regeneration in rat femurs compared to non-polarized PVDF [169,214].

## 5. Future Challenges

While contemporary tissue engineering methods have progressed significantly, there remain numerous unresolved limitations and challenges within the field, including the following.

- Effective integration of piezoelectric nanomaterials into existing bone tissue. Issues related to biomechanics and biomechanical compatibility may affect the durability and effectiveness of piezoelectric stimulation in physiological conditions.
- In vivo studies on piezoelectric scaffolds are full of challenges, such as physiological differences between animals and humans, material degradation issues, post-implant inflammation, and incomplete knowledge of bioelectric mechanisms. Further research is needed to meet these requirements.
- The biological environment in organisms is very complex. The challenge is to understand how piezoelectric nanomaterials behave in a dynamic cellular environment, taking into account various aspects, such as movement, fluid flow, and the presence of other cells.
- The immune system’s response to the introduction of nanomaterials may pose a challenge. Potential side effects, immunological reactions, and long-term consequences of the use of piezoelectric nanomaterials in bone tissue must be taken into account.
- Obtaining piezoelectric materials with appropriate mechanical, electrical, and biocompatibility properties can be a challenge. It is also important that these materials are available in sufficient quantities for the potential scale of application in therapeutics and tissue engineering.
- The introduction of new technologies must take into account ethical issues as well as issues related to patient safety. Research into the long-term effects and potential risks to patient health is essential.

## 6. Conclusions

Research on the use of piezoelectric nanomaterials in tissue engineering shows the promising potential of piezoelectric stimulation in the process of bone regeneration. In vitro and in vivo experiments provide evidence of the beneficial effects of these materials on the proliferation and differentiation of bone cells [82]. However, despite promising results, there are limitations related to the effective integration of piezoelectric nanomaterials into the biological environment of bone tissue. Further understanding of cellular interactions, biomechanics, and the long-term effects of stimulation is needed. One of the answers is the use of piezoelectric stimulation, which can accelerate the healing process of bone fractures and support the formation of a dense bone tissue structure. This opens up prospects for shortening the recovery time of patients and improving the effectiveness of treatment of bone injuries.

In the context of future research, it is worth considering the role of artificial intelligence in optimizing the applications of piezoelectric nanomaterials. Machine learning algorithms can support the design of materials with optimal biomechanical and biological properties.

In order to transfer this promising research from the laboratory to clinical practice, further clinical studies are necessary to evaluate the effectiveness, safety, and long-term results of the use of piezoelectric nanomaterials in bone regeneration therapy.

In the context of future research, it is necessary to focus on research on the long-term safety of using piezoelectric nanomaterials in organisms while taking into account potential side effects and the immune system’s response.

## Figures and Tables

**Table 1:** Piezoelectric coefficient, relative permittivity, and conductivity of bone and articular cartilage.

	Bone		Articular Cartilage	Ref.
	Cortical	Trabecular		
Piezoelectric Coefficient (d_33_) [pC/N^−1^]	0.7–2.3		0.2–0.7	[16]
Relative Permittivity	1.45 × 10^2^	2.49 × 10^2^	1.39 × 10^3^	[17]
Conductivity (S/m)	0.02	0.079	1.14 ± 0.11	[18]

**Table 2:** The piezoelectric coefficient for different natural piezoelectric materials.

Type of Material	Piezoelectric Materials	Piezoelectric Coefficient	Ref.
Polysaccharides	Chitosan	d_33_ = 18.4 pCN^−1^	[47]
	Chitin (nanofibers)	d_33_ = 9.49 pCN^−1^	[48]
	Cellulose	d_33_ = 19.3 ± 2.9 pCN^−1^	[49]
Proteins	Collagen	d_14_ = 12 p N^−1^	[50]
	Keratin	d_14_ = 1.8 pCN^−1^	[51]
	Silk	d_14_ = 5–1.5 pCN^−1^	[52]
	Fish swim bladder (FSB)	d_33_ = 22 pCN^−1^	[53]

**Table 3:** Natural piezoelectric materials and their applications in bone tissue engineering.

Materials	Advantages	Description and Application	Ref.
Chitosan	-biocompatible -antibacterial -biodegradable -high porosity -non-cytotoxic	Bone tissue engineering	[72]
		Chitosan/alginate hydrogels containing parathyroid hormone (PTH), peptide, and hydroksyapatite (HA) dedicated to cranial bone regeneration.	[73]
		Chitosan-coated poly(trimethylene carbonate)/oleic-acid-treated HA/ PLA/vancomycin hydrochloride microsphere scaffold used in BTE.	[74]
Cellulose	-biocompatible -non-cytotoxic -high tensile strength -biodegradable	A cellulose porous scaffold was prepared through incorporation of paraffin wax with bacterial cellulose via a fermentation process for a higher osteoblast response.	[58]
Keratin	-structural integrity -biocompatibility -biodegradability -bioactivity	Keratin-based biomaterials in orthopedic tissue engineering.	[75]
		Electrospun poly(3-hydroxybutyrate)/keratin scaffold for bone tissue engineering focused on the osteogenic activity of the scaffolds, which is enhanced in the presence of keratin.	[76]
		Silk fibroin/wool keratin composite scaffold with a hierarchical fibrous and porous structure formed through electrospinning with potential use in meniscal repair and healing bone injuries.	[77]
Collagen	-biocompatibility -biodegradability -bioactivity	Preparation of 3D porous microsphere of collagen/BMP-2/bacterial cellulose through the reverse-phase suspension regeneration method to promote biocompatibility, osteogenic differentiation, proliferation, and adhesion of mice cells in 3D scaffolds.	[78]
		Composites made from the piezoelectric component of bone, collagen (fibrilar bovine collagen type I), used to fabricate materials for bone substitutes.	[79]
		A scaffold combination of collagen and hydroxyapatite that exhibits osteoconductive properties.	[80]

**Table 4:** The piezoelectric coefficient for different synthetic piezoelectric materials.

Type of Material	Piezoelectric Materials	Piezoelectric Coefficient	Ref.
Polymers	Polyvinylidene fluoride (PVDF)	d_31_ = 23 pCN^−1^	[78]
	Polyvinylidene fluoride–trifluoroethylene (PVDF-TrFE)	d_33_ = 38 pCN^−1^	[79]
	Poly (l-lactic acid) (PLLA)	d_14_ = 9.82 pCN^−1^	[83]
	Polyhydroxybutyrate (PHB)	d_14_ = 1.6–2 pCN^−1^	[14]
	Poly(3-hydroxybutyrate-co-3-hydroxyvalerate) (PHBV)	d_14_ = 1.3 pCN^−1^	[14]
	Glycine–polyvinyl alcohol (PVA)	d_33_ = 5.3 pCN^−1^	[84]
Peptide	Poly-γ-benzyl-L-glutamate (PBLG)	d_33_ = 25 pCN^−1^	[85]
	Poly-γ-methyl-L-glutamate (PMLG)	d_14_ = 2 pCN^−1^	[83]

**Table 5:** Synthetic piezoelectric materials and their applications in bone tissue engineering.

Materials	Advantages	Description and Application	Ref.
Polyvinylidene fluoride (PVDF)	-easy to process -high piezoelectric coefficient -non-cytotoxic -flexible -biocompatible	Application in bone tissue engineering.	[27]
		PVDF scaffold largely promoted the osteogenic differentiation of human-adipose-derived stem cells.	[167]
		Actuator device based on PVDF with effective stimulation properties for bone growth.	[168]
		Bone formation in vitro.	[95]
		Osteogenic differentiation in vitro.	[169]
		In vivo tests of β-PVDF polymer samples were conducted through implantation in rats’ bones.	[170]
		Significant increase in cell viability compared to control samples.	[171]
Polyvinylidene fluoride trifluoroethylene (PVDF-TrFE)	-high piezoelectric coefficient -flexible -non-cytotoxic -biocompatible	Faster bone regeneration and higher osteogenic properties of bone cells.	[172]
		Piezoelectric fibers promoted the MSCs’ chondrogenic differentiation and the osteogenic differentiation of MSCs.	[173]
		Cartilage tissue engineering.	[83]
		Bone, cardiac, neural, skin TE, cartilage.	[14,174,175]
Poly (l-lactic acid) (PLLA)	-biocompatible -elastomeric behavior -biodegradable -non-cytotoxic -corrosion resistance -easy to process	In vitro PLLA blends for bone regeneration.	[176]
		Bone regeneration in vivo.	[177]
		Electrospun P(LLA-CL) type I collagen for BTE.	[178]
		Electrospun PLLA with freeze-dried collagen promotes the osteogenic differentiation of seeded MSCs in vitro. In vivo tests on damaged rabbits’ bones enhanced AC formation.	[179]
		Electrospun PLLA, PCL, and PLLA/PCL scaffolds were seeded with MSCs. All scaffolds promoted the chondrogenic differentiation of MSCs.	[180]
		Cartilage, vascular, medical devices (e.g., screws), skin, bone, and neural TE, wound dressing, and drug delivery.	[72,181,182]
PHB/PHBV	-biodegradable -biosynthesized -resistant to UV radiation -low moisture permeability -good moisture resistance -provides an odor barrier	Proliferation and differentiation of rabbit bone marrow cells.	[183]
		Multiple applications in BTE.	[69]
		Higher cell proliferation and differentiation on PHB/HA scaffolds compared to the PHB samples.	[184]

## Data Availability

No new data were created or analyzed in this study. Data sharing is not applicable to this article.

## Abbreviations

**Abbreviation**	**Full Name**
ALP	alkaline phosphatase
ATDC5	clonal mouse embryonic cell line
BMP-2	bone morphogenetic protein 2
BMSCs	bone marrow mesenchymal stem cells
BTE	bone tissue engineering
CaP	calcium phosphate
Ca-P-Si	calcium phosphate silicate
CNT	carbon nanotubes
DPSCs	dental pulp stem cells
ECM	extracellular matrix
EMF	electromagnetic field
ES	electrical stimulation
FDM	fused deposition modeling
FSB	fish swim bladder
GGMA	methacrylated gellan gum
GO	graphene oxide
HA	hydroksyapatite
hBMMSCs	human-bone-marrow-derived mesenchymal stem cells
IL-1	interleukin-1
iPSCs	induced pluripotent stem cells
MAPK	mitogen-activated protein kinase
mBMSCs	mouse bone marrow stromal stem cells
MSCs	mesenchymal stem cells
MWCNT	multiwalled carbon nanotube
NF-AT	nuclear factor of activated T-cells
PANI	polyaniline
PA-11	polyamide-11
PBLG	poly-γ-benzyl-L-glutamate
PCL	polycaprolactone
PCL-TCP	polycaprolactone–tricalcium phosphate
PDA	perylene-3, 4,9, 10-tetracarboxylic dianhydride
PEMF	pulsed electromagnetic field
PHB	poly(3-hydroxybutyrate)
PHBV	poly(3-hydroxybutyrate-co-3-hydroxyvalerate)
PKC	protein kinase C
PLA	poly(lactic acid)
PLLA	poly (l-lactic acid)
PMLG	poly-γ-methyl-L-glutamate
PMs	piezoelectric materials
PPy	polypyrrole
PTH	parathyroid hormone
PVA	polyvinyl alcohol
PVDF	polyvinylidene fluoride
PVDF-HFP	poly(vinylidene fluoride-hexafluoropropylene)
PVDF-TrFE	polyvinylidene fluoride-trifluoroethylene
RGD	l-arginyl-glycyl-l-aspartic acid sequence
ROS	reactive oxygen species
SBF	simulated body fluid
SLS	selective laser sintering
SMs	smart materials
TCP	tricalcium phosphate
TE	tissue engineering
US	ultrasound
ZIF-8	zinc-based metal–organic frameworks
